# Quantifying Gamma-Interferon from CMV-Specific CD8+ T Cells Defines Protection Against Clinically Significant CMV Infection in Solid Organ Transplant Recipients: The Quanti-CMV Score

**DOI:** 10.3390/microorganisms13030589

**Published:** 2025-03-04

**Authors:** Elisa Ruiz-Arabi, Juan José Castón, Aurora Páez-Vega, Raquel Fernández-Moreno, Federico Giovagnorio, Belén Gutiérrez-Gutiérrez, Angela Cano, Alberto Rodríguez-Benot, José M. Vaquero-Barrios, Isabel Machuca, Elisa Vidal, Sara Cantisán, Julián Torre-Cisneros

**Affiliations:** 1 Service of Infectious Diseases, Reina Sofia University Hospital, 14004 Córdoba, Spain; elisa.ruiz.arabi.sspa@juntadeandalucia.es (E.R.-A.); angela.cano.sspa@juntadeandalucia.es (A.C.); isabelm.machuca.sspa@juntadeandalucia.es (I.M.); elisa.vidal.sspa@juntadeandalucia.es (E.V.); julian.torre.sspa@juntadeandalucia.es (J.T.-C.); 2 Maimonides Institute for Biomedical Research of Cordoba (IMIBIC), Reina Sofia University Hospital, University of Cordoba (UCO), 14004 Córdoba, Spain; aurora.paez@imibic.org (A.P.-V.); raquelfm2@gmail.com (R.F.-M.); alberto.rodriguez.benot.sspa@juntadeandalucia.es (A.R.-B.); vaquerosenior@gmail.com (J.M.V.-B.); sara.cantisan.sspa@juntadeandalucia.es (S.C.); 3 Centro de Investigación Biomédica en Red de Enfermedades Infecciosas (CIBERINFEC), Instituto de Salud Carlos III, 28029 Madrid, Spain; belengutiguti@hotmail.com; 4 Department of Molecular Medicine, University of Padua, 35121 Padua, Italy; federico.giovagnorio@studenti.unipd.it; 5 Infectious Diseases Service, Virgen Macarena University Hospital, University of Seville, Biomedicine Institute of Seville (IBIS), 41009 Seville, Spain; 6 Department of Nephrology, Reina Sofía University Hospital, 14004 Córdoba, Spain; 7 Lung Transplantation Unit, Reina Sofia University Hospital, 14004 Córdoba, Spain; 8 Immunology Service, Reina Sofia University Hospital, 14004 Córdoba, Spain

**Keywords:** cytomegalovirus, SOT, transplantation, infection, kidney, lung, QuantiFERON-CMV, cell-mediated, immunity, cutoff

## Abstract

The cutoff value of the commercial interferon (IFN)-γ release assay (QuantiFERON-CMV) proposed by the manufacturer is assumed to be predictive. We aimed to determine the optimal cutoff value for protection against clinically significant cytomegalovirus (CMV) infection within 30 days. We analyzed two different cohorts: adult CMV seropositive kidney transplant (KT) recipients with antithymocyte globulin (ATG) induction from the TIMOVAL study and seropositive lung transplant (LT) patients from the CYTOCOR study. The optimal cutoff value was established using Youden’s index. We estimated the predictive capacity of the cutoff value through the AUROC and assessed the diagnostic accuracy of the assay at the different cutoff values. We finally evaluated clinical variables that could improve the predictive ability of the assay on a predictive score. Four hundred-four samples from 130 transplant recipients were analyzed. The optimal cutoff value was ≥2.2 IU/mL for both populations, with a positive predictive value of 99% and 99.5% (95% CI, 98–100%) for KT and LT recipients, respectively. The AUROC of the predictive score was 0.85 (95% CI, 0.73–0.97). Using the proposed cutoff value and the Quanti-CMV score may allow the individualization of preventive strategies and serve as an objective tool to support clinical decision-making.

## 1. Introduction

Cytomegalovirus (CMV) infection after solid organ transplantation (SOT) remains one of the leading causes of mortality and morbidity in this population [1]. Despite the growing evidence of the effectiveness of CMV-specific cell-mediated immunity (CMV-CMI) in preventing CMV infection and disease [2,3,4,5,6], its adoption in transplant centers remains limited. This is partly due to the absence of a consensus on the optimal technique for implementation [7]. While enzyme-linked immunosorbent spot (ELISpot) assays and intracellular cytokine staining (ICS) assays with flow cytometry may provide more detailed immunological information, they require specialized equipment and expertise, and most importantly, they lack a cutoff value and/or technical standardization [8,9]. By contrast, the QuantiFERON^®^-CMV (QF-CMV) test (Qiagen GmbH) is a commercial enzyme-linked immunosorbent assay (ELISA)-based interferon (IFN)-γ release assay that benefits from feasibility, technical simplicity, and validated cutoff values [10]. Moreover, its potential for complete automation [11] sets it apart from other techniques for clinical laboratory application.

Observational studies comparing the different techniques have observed a tendency toward a poorer predictive capacity of the QF-CMV test [12,13,14,15,16,17,18]. However, they stand out for their heterogeneity as most of them use the cutoff value provided by the manufacturer (≥0.2 IU/mL (CMV minus nil)) [19]. While the optimal predictive cutoff value is being evaluated in the other techniques, as it is not standardized, the manufacturer’s cutoff value has been generally assumed to be predictive in the case of QF-CMV. Few studies have previously investigated the optimal predictive cutoff value for the QF-CMV technique [20,21,22,23]. These studies suggest that increasing the cutoff value in CMV-seropositive recipients could improve the test’s predictability, making it comparable to other methods [15] and even becoming a cost-effective strategy [23]. Nevertheless, these studies show variability in the population studied and in the event to be predicted (disease vs. infection). Given the diverse range of immunosuppression regimens employed, the various types of transplanted organs, and the varying serostatuses of donors and recipients, the optimal cutoff value may differ in each clinical scenario. Additionally, the ideal timing after transplantation to perform CMV-CMI has not yet been established [7,24].

Considering that the predictive capacity of the QF-CMV assay depends on the particular IFN-γ production of each patient during the posttransplant period, the present observational study aimed to determine the optimal cutoff value for predicting protection from clinically significant CMV infection (CS-CMV) within 30 days after QF-CMV determination.

## 2. Materials and Methods

### 2.1. Study Population and Setting

Adult (≥18 years) patients who underwent a kidney transplant (KT) or a lung transplant (LT) from two different multicenter, randomized controlled trials (RCTs) were selected. KT recipients came from the TIMOVAL study [2] and LT patients from the CYTOCOR study [25] (NCT03699254). Both studies shared an experimental group where immunoguided prophylaxis was carried out by serial QF-CMV determination. All patients had a positive pre-transplant CMV serology. KT patients from the TIMOVAL study received antithymocyte globulin (ATG) induction. They were enrolled between August 2016 and October 2018. LT patients from the CYTOCOR study were enrolled between April 2019 and May 2023. The present study is a subanalysis of two RCTs approved by the coordinating hospital’s ethics committee (Reina Sofia University Hospital), following the guidelines of Good Clinical Practice (Helsinki Declaration) and applicable Spanish law. All the data collected were anonymized. Both trials were registered in EudraCT (2015-004406-42; 2018-003300-39).

### 2.2. Study Design and Outcomes

In the TIMOVAL study [2], patients underwent QF-CMV determination at days 30, 45, 60, and 90 after transplantation, with the discontinuation of prophylaxis when positive CMV-CMI (QF-CMV ≥ 0.2 IU/mL (CMV minus nil)) was achieved. All patients received at least 30 days of prophylaxis and were followed up for 12 months. Meanwhile, in the CYTOCOR cohort [25], patients received a monthly determination of QF-CMV from months 3 to 12 after transplantation, with the discontinuation of prophylaxis when positive QF-CMV (≥0.2 IU/mL (CMV minus nil)) was reached. All CYTOCOR patients received at least 90 days of prophylaxis and were followed up for 18 months. CMV viral load was determined at least every two weeks in both cohorts. For the present study, QF-CMV determination followed by the initiation of antiviral treatment before viral load determination was excluded. The study outcome was the occurrence of CS-CMV within 30 days after QF-CMV determination in patients without antiviral prophylaxis. We also assessed clinical variables that could improve the predictive ability of the QF-CMV assay on a predictive score. These variables were retrospectively collected using a standardized case report form.

### 2.3. Laboratory Methods

The measurement of CMV-CMI using the QF-CMV assay was performed according to the manufacturer’s instructions [19]. Briefly, one milliliter of heparinized whole blood was collected in three QF collection tubes. The tubes contained a mix of 22 CMV peptides (“CMV tube”), a negative control with no antigens (“nil tube”), or a positive mitogen control (phytohemagglutinin) (“mitogen tube”). After 16–24 h of incubation at 37 °C, supernatants were harvested and analyzed for IFN-γ (IU/mL) using ELISA. IFN-γ levels were interpreted as follows: (a) reactive QF-CMV assay at ≥0.2 IU/mL (CMV minus nil tubes) and ≥25% of the nil tube; (b) nonreactive assay at <0.2 IU/mL (CMV minus nil) and/or <25% of the nil tube, as well as ≥0.5 IU/mL (mitogen minus nil); and (c) indeterminate assay at <0.2 IU/mL (CMV minus nil) and/or <25% of nil, as well as <0.5 IU/mL (mitogen minus nil). For the purpose of our study, the results of the technique were considered from a quantitative rather than a qualitative perspective. Therefore, the quantitative value of each sample was obtained from the value in the “CMV tube” minus the value in the negative control “nil tube”. The QF-CMV indeterminate results were also considered as nonreactive (0.0 IU/mL).

Plasma CMV load was analyzed via real-time polymerase chain reaction (RT-PCR) using the technique implemented at each center. Samples available in each laboratory were analyzed and carried out according to the protocol and by indication of the responsible physician. The pre-transplant CMV serostatus was determined using the specific anti-CMV IgG ELISA routinely used in each center’s laboratory.

### 2.4. Definitions

CS-CMV was defined as the threshold of CMV DNAemia for initiating preemptive antiviral therapy in asymptomatic patients (>1500 IU/mL in plasma) or the occurrence of CMV disease. The diagnosis of CMV disease included both “end-organ disease” and “CMV syndrome” [26]. In this study, the term “sex” refers to “sex assigned at birth”, as recorded in the standardized case report form.

### 2.5. Statistical Analysis

Results were expressed as the median and interquartile range (IQR) for continuous variables and as absolute and relative frequencies for categorical variables. Continuous variables were compared using Student’s *t* test or the Mann–Whitney *U* test. Categorical variables were compared using the χ^2^ test or Fisher’s exact test. The optimal cutoff value was established using Youden’s index (J = sensitivity + specificity − 1) [27]. Time-to-event curves were plotted using the Kaplan–Meier method, and intergroup differences were compared with the log-rank test. The predictive capacity of the different cutoff values was estimated through the area under the receiver operating characteristic curve (AUROC). The DeLong test was employed to compare differences. The diagnostic accuracy of the QF-CMV assay at the different cutoff values was evaluated through sensitivity, specificity, positive predictive values (PPVs), and negative predictive values (NPVs) with 95% confidence intervals (CIs). We analyzed variables associated with the study outcome using univariate logistic regression analysis. Classification and regression tree (CART) analysis was used to support the choice of the final multivariate model. Weighted scores for each variable were calculated by dividing each regression coefficient by one-half of the smallest coefficient and rounding them to the nearest integer. All the significance tests were two-tailed. QF-CMV indeterminate results were also considered nonreactive (0.0 IU/mL) for the analysis. The SPSS version 25.0 software, R software (version 4.3.1), and CART software 8.3 were used for the analysis.

## 3. Results

### 3.1. Characteristics of the Study Population

A total of 404 samples from 130 transplant recipients were analyzed (71 KT and 59 LT recipients). One hundred thirty-five samples came from KT recipients and two hundred sixty-nine from LT recipients. Clinical characteristics are shown in Table 1. In the month before QF-CMV determination, 129 (31.9%) samples received antiviral prophylaxis, and 84 (20.8%) showed CMV replication at any level. The median number of days posttransplantation for QF-CMV determination in KT and LT recipients was 90 (IQR: 60–90) and 240 (IQR: 150–300) days, respectively. No LT recipients received ATG induction. The median QF-CMV value was 4.7 IU/mL (IQR: 1.1–18.6) in KT recipients and 10.8 IU/mL (IQR: 2.0–42.3) in LT recipients. There were two indeterminate results, both in KT recipients. Ninety samples (22.3%) showed CMV replication in the month following QF-CMV determination. There were seventeen (4.2%) CS-CMV cases, twelve from KT and five from LT, including one CMV disease as a viral syndrome in an LT recipient. The samples with CS-CMV were all from different patients.

### 3.2. Evaluation of the Best Predictive Cutoff Value

**Figure 1:**
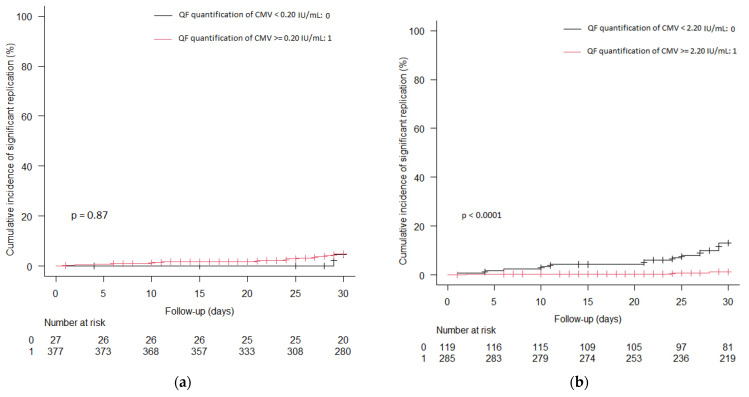
Cumulative hazard curves of clinically significant CMV infection according to different criteria for QF-CMV assay positivity based on IFN-γ levels (CMV minus nil): (**a**) cutoff value proposed by the manufacturer (≥0.2 IU/mL) (log-rank *p* = 0.87) and (**b**) optimal cutoff value (≥2.2 IU/mL) (log-rank *p* < 0.0001). QF quantification of CMV < 0.2 IU/mL and <2.2 IU/mL includes nonreactive and indeterminate results. CMV, cytomegalovirus; QF, QuantiFERON.

We explored the best predictive cutoff value for CS-CMV in the month after QF-CMV determination using Youden’s Index [27]. To determine whether the cutoff value varied according to the type of transplanted organ, KT and LT recipients were analyzed separately. The optimal cutoff value was ≥2.16 IU/mL for both KT and LT recipients, which we rounded to ≥2.2 IU/mL. We presented cumulative hazard curves to illustrate the probability of CS-CMV by comparing samples with a QF-CMV determination value higher versus lower than the manufacturer’s established cutoff value (0.2 IU/mL) (Figure 1a) and the cutoff value obtained from our analysis (2.2 IU/mL) (Figure 1b). There were no significant differences in the cumulative incidence of CS-CMV (*p* = 0.87) between QF-CMV ≥ 0.2 and <0.2, in comparison with QF-CMV ≥ 2.2 and <2.2 (*p* < 0.0001). These differences were also observed when we analyzed KT and LT recipient samples separately (Figure S1).

The new cutoff value also showed an AUROC for CS-CMV in KT recipients of 0.82 (95% CI: 0.71–0.92) compared with an AUROC for the manufacturer’s recommended cutoff value of 0.50 (95% CI: 0.33–0.67). These differences in AUROCs were significant (*p* < 0.05). The proposed cutoff value in LT recipients showed an AUROC of 0.78 (95% CI: 0.51–1) compared to the manufacturer’s recommended value of 0.47 (95% CI: 0.2–0.74). These differences in AUROCs were also significant (*p* < 0.05). Table 2 shows the characteristics of the samples according to the optimal cutoff value obtained (<2.2 IU/mL and ≥2.2 IU/mL). In samples with a QF-CMV determination value of <2.2 UI/mL, 12% had significant CS-CMV within 30 days, in contrast with 1% of samples with a QF-CMV assay value of ≥2.2 UI/mL (*p* < 0.001). CMV replication was also significantly higher at any level in the <2.2 UI/mL group. There were no other significant differences between the groups. Figure S2 represents the posttransplant kinetics of QF-CMV in LT and KT recipients.

To further investigate the impact of ATG induction on the predictive capacity of the test, we compared the time interval from the administration of ATG to the determination of QF-CMV between KT recipients who developed CS-CMV and those who did not, revealing no significant differences (*p* = 0.36). Similarly, the median ATG induction dose was not significantly different between those diagnosed with CS-CMV (4.03 mg/kg [IQR: 1.93–6.67]) and those who did not develop CS-CMV (3.69 mg/kg [IQR: 2.82–4.59]) (*p* = 0.54).

#### Comparison with the Prior Cutoff Value in KT Recipients

We also conducted a comparison of the predictive capacity associated with the cutoff value established in our study against that proposed by the sole prior study conducted on KT recipients treated with ATG [22]. The new cutoff value showed an AUROC for CS-CMV of 0.82 (95% CI: 0.71–0.92) compared with an AUROC for the cutoff value proposed by Fernández-Ruiz et al. [22] (≥1.13 IU/mL) of 0.62 (95% CI: 0.45–0.80). These differences in AUROCs were significant (*p* < 0.05).

### 3.3. Diagnostic Accuracy Using the Optimal Cutoff Value

We compared the diagnostic accuracy of the test for predicting protection from CS-CMV out to 30 days post-QF-CMV determination, using the cutoff value proposed by the manufacturer (≥0.2 IU/mL) and the optimal cutoff value obtained via Youden’s Index (≥2.2 IU/mL). We calculated sensitivity, specificity, PPV, and NPV for the different cutoff values in KT (Table 3a) and LT recipients (Table 3b). Using the optimal cutoff value in KT recipients, the test’s specificity increased to 92% (95% CI, 84–100%), with a PPV of 99% (95% CI, 98–100%) for protection from CS-CMV. Similarly, the test’s specificity in LT recipients rose to 80% (95% CI, 45–100%), with a PPV of 99.5% (95% CI, 98–100%).

### 3.4. Calculation of a New Predictive Score for CS-CMV (Quanti-CMV Score)

The univariate logistic regression analysis for developing CS-CMV is shown in Table S1. Based on variables that demonstrated significant association, CART analysis was used to support the choice of the final multivariate model (Figure S3). This model showed an AUROC of 0.86 (95% CI, 0.74–0.98). Considering these three variables, we finally developed a CS-CMV predictive score (Table 4). Based on their beta coefficient, we assigned a score of three to QF-CMV determination <2.2 IU/mL and a score of two to induction therapy with ATG and any level of CMV replication the month before QF-CMV determination, respectively. The final AUROC of the score was 0.85 (95% CI, 0.73–0.97). We determined sensitivity, specificity, PPV, and NPV for the different score values (Table 5). In our cohort, 34.9% of samples presented a score of ≥3, showing a sensitivity of 88.2% (95% CI, 68–100%), 67.4% (95% CI, 62.8–72%) specificity, 99.2% (95% CI, 98.1–100%) NPV, and 10.6% (95% CI, 5.6–15.6%) PPV. We compared the cumulative hazard curves of CS-CMV according to this breakpoint (<3 and ≥3) (*p* < 0.0001; Figure S4). A maximum score of seven points achieved a PPV for CS-CMV of 40% (95% CI, 15.2–64.8%), with a specificity of 97.7% (95% CI, 96.2–99.1%) and an accuracy of 95% (95% CI, 92.9–97.1%).

## 4. Discussion

The present study is the first to determine the best cutoff value for the QF-CMV assay in predicting protection from CS-CMV within 30 days after QF-CMV determination in seropositive KT recipients with ATG induction and seropositive LT recipients, separately. In light of the results, the predictive capacity of the assay may be improved by modifying the cutoff value established by the manufacturer to define positivity. This cutoff value would be the same in two distinct populations with a moderate risk of CMV infection, each employing slightly different preventive strategies [7]. Furthermore, the predictive capacity of the technique may be enhanced by incorporating clinical variables into a predictive score.

Increasing the manufacturer’s cutoff value from ≥0.2 IU/mL to ≥2.2 IU/mL improves the predictive capacity of the technique, and this value is consistent for both seropositive KT recipients with ATG induction and seropositive LT recipients. As shown in the cumulative hazard curves, there were no significant differences in the cumulative incidence of CS-CMV (*p* = 0.87) between QF-CMV ≥ 0.2 and <0.2, in comparison with QF-CMV ≥ 2.2 and < 2.2 (*p* < 0.0001). The new cutoff value also showed an increase in the AUROC for CS-CMV compared to the manufacturer’s recommended cutoff value in both populations (0.82 vs. 0.50 in KT recipients and 0.78 vs. 0.47 in LT recipients). Our results also indicate that the time elapsed since the induction with ATG and its dosage did not significantly influence the predictive capacity of the test utilizing this newly established optimal cutoff value.

The new cutoff value (≥2.2 IU/mL) improved the PPV for protection against CS-CMV to 99% and 99.5% in renal and lung transplant recipients, respectively. Notably, the test’s specificity increased from 8% to 92% using the new cutoff value in KT recipients and from 0% to 80% in LT recipients. However, the NPV remained low despite increasing the cutoff value (24% in KT recipients and 5.7% in LT recipients). These results confirm the usefulness of the QF-CMV technique in predicting protection, although it is ineffective in predicting CS-CMV in its absence, as other factors must contribute to that risk [28].

Developing a predictive score by adding clinical variables enhanced the diagnostic accuracy for CS-CMV. This model showed an AUROC of 0.85 (95% CI, 0.73–0.97), a value higher than the optimal QF-CMV cutoff value alone in kidney (0.82 [95% CI: 0.71–0.92]) and lung transplant recipients (0.78 [95% CI: 0.51–1]). The score also exhibited high specificity and NPV. However, although the PPV for CS-CMV increased, its maximum value, with a score of seven, was 40%, with a specificity of 97.7% and an accuracy of 95%. Additionally, only 3.7% of samples reached this maximum score. It is noteworthy that, despite efforts to enhance the predictive capacity for CS-CMV risk, the score again exhibited higher NPVs than PPVs, which means that it is useful for predicting protection against CS-CMV, not the risk of developing it. By selecting a cutoff score of <3, which could be achieved with a QF-CMV value of ≥2.2 IU/mL, or by not having viral replication in the month before QF-CMV determination and not receiving ATG as induction therapy, we could predict the absence of CS-CMV with 99.2% certainty. This score was present in almost 35% of the study sample. In line with these results, the cumulative hazard curves of CS-CMV according to this breakpoint (<3 and ≥3) also showed statistically significant differences.

The optimal QF-CMV cutoff value proposed (≥2.2 IU/mL) differs from those previously described by Fernández-Ruiz et al. [22] (≥1.13 IU/mL), Kumar et al. [20] and Lisboa et al. [21] (≥0.1 IU/mL), and Reusing et al. [23] (≥1.0 IU/mL). It is important to note that these studies are not directly comparable as they measure different outcomes, such as CMV infection [22], CMV disease [20], or virologic and/or clinical progression [21]. Moreover, many of these studies are conducted across various populations, each with distinct risks associated with the development of CMV disease [23]. This variability may account for the differences observed in the cutoff values. Studies that involve a combination of different types of transplanted organs and serological profiles contribute to a noticeable imbalance in the risk of CMV disease within the studied population [20,21]. To our knowledge, no research has explicitly investigated the optimal predictive cutoff value for QF-CMV in lung transplant patients. Interestingly, in the RCT carried out by Westall et al. [4], they described a QF-CMV mean value in the immunoguided arm at five and eight months posttransplantation of 2.2 IU/mL and 2.8 IU/mL (standard error [SE] of 0.4), respectively. This value is close to our cutoff, with a median posttransplant time of eight months. Although the study conducted by Fernández-Ruiz et al. [22] aimed to predict protection from CMV infection, not CS-CMV, we compared the predictive capacity of their previously established cutoff value (≥1.13 IU/mL) with our optimal cutoff value through the AUROC. The AUROC associated with the former value was lower than the one obtained using our calculated cutoff value. Reusing et al. [23] used the same CS-CMV outcome [23], although in a lower-risk population, KT recipients without ATG induction, which would explain the lower cutoff value obtained (≥1.0 IU/mL). Finally, even though the diagnostic accuracy of the assay is not directly comparable to other studies due to differences in the predicted outcome, recipient serostatus, and the type of transplanted organ [29], we observed that increasing the cutoff value improves specificity and PPV [21,22]. 

We recognize that using the term ‘CS-CMV’ entails a degree of subjectivity and is not widely accepted as an endpoint in SOT [26]. Nevertheless, in light of its growing adoption in recent clinical trials [3,23], we believe that this definition more accurately represents standard clinical practice. Moreover, our study implemented a threshold for asymptomatic viral replication in the plasma (>1500 IU/mL), which aligns with the recommended threshold for high-risk transplant recipients [30]. Given the potential for spontaneous clearance, the clinical relevance of predicting any level of viral replication remains questionable [21]. Additionally, considering the low incidence of CMV disease within this population [2,3,4,31,32], predicting this event would require laborious studies with larger sample sizes, making it almost unfeasible.

The present study has some limitations. The low percentage of CS-CMV observed may be attributable to a survival bias, as samples with antiviral prophylaxis following QF-CMV determination were excluded, i.e., those with a nonreactive result (<0.2 IU/mL) in the assay. Consequently, we may have underestimated the number of patients at higher risk of CS-CMV during the early posttransplant period, where a nonreactive result is more likely. This is reflected in the median posttransplant days for KT and LT recipients at 90 and 240, respectively. Furthermore, since consecutive samples were used, if we examine the incidence of CS-CMV based on the total percentage of patients, the results are similar to those previously reported [2,3,4]. It is important to note that all samples that developed CS-CMV originated from different patients. This ensures that our results are not overestimated due to the use of samples rather than individual patients. Secondly, given that no LT recipient received ATG induction, the additional value of the predictive score, compared to the QF-CMV cutoff alone, is limited in this population. In this context, the predictive score would be particularly relevant in centers lacking access to CMV-CMI techniques. Finally, our results require external validation. We faced several limitations in validating our QF-CMV score in an external cohort. These include the low percentage of patients at our center who had undergone a QF-CMV test and discontinued antiviral prophylaxis within the following 30 days, particularly among LT recipients, where immunoguided prophylaxis was not yet implemented when this study was conducted. The proportion of those treated with ATG induction and CS-CMV incidence among KT patients was also insufficient. Lastly, the lack of standardization in defining CS-CMV as >1500 IU/mL in the plasma for asymptomatic patients before 2019 in our center resulted in some patients being treated for lower viral loads. Moreover, although the predictive score has been explicitly studied in these defined populations, it is intended to be applicable across different organ types, which also requires validation. 

One of the study’s strengths regarding developing the predictive score is that we controlled for potential confounding variables by conducting the multivariate CART analysis. The median number of posttransplantation days at which QF-CMV determination was made (90 days for KT recipients with ATG induction and 240 days for LT recipients) aligns with the recommended timeframes for transitioning to preemptive therapy in these populations [7], which enhances its applicability in clinical practice. In light of our results, applying the new cutoff value would enable the extension of CMV surveillance during preemptive therapy to every 30 days instead of weekly viral load monitoring, thereby reducing costs [23], facilitating implementation, and optimizing the preemptive strategy. Moreover, the predictive score would be particularly relevant in centers implementing preemptive therapy without access to CMV-CMI techniques. Given that the absence of viral replication in the preceding month and the lack of ATG induction therapy could predict the absence of CS-CMV with 99.2% certainty, this approach may enhance clinical decision-making. In centers that follow prevention protocols based on universal prophylaxis, both the optimal cutoff value and the predictive score could aid in determining the appropriate timing for discontinuing prophylaxis due to their ability to predict protection against CS-CMV.

In summary, our results demonstrate that the quantitative value of the QF-CMV test is more reliable than the standardized qualitative value in predicting protection against CS-CMV. Moreover, the optimal cutoff value may vary depending on the clinical scenario. Implementing the proposed cutoff value and the Quanti-CMV score in the studied population could facilitate the individualization of preventive strategies and serve as an objective tool to support clinical decision-making. Consequently, it may enable longer intervals between viral load surveillance during preemptive therapy, reducing monitoring costs and improving feasibility in clinical practice. Furthermore, these findings could help ensure the safe discontinuation of valganciclovir in patients receiving universal prophylaxis. While external validation and comparative studies between the various CMV-CMI techniques using the new cutoff value are needed, assessing its applicability in other transplanted organs is also essential.

## Figures and Tables

**Table 1:** Demographics and clinical characteristics of the study samples (*n* = 404).

Variable	
Age, years, median (IQR)	61.0 (55.0–65.0)
Sex	
Male	255 (63.1)
Type of transplant	
Kidney transplant	135 (33.4)
Lung transplant	269 (66.6)
Induction therapy	
ATG ^a^	135 (33.4)
Basiliximab	202 (50.0)
Primary immunosuppression regimen	
Steroids	404 (100.0)
Tacrolimus	404 (100.0)
mTOR inhibitor (Everolimus)	14 (3.5)
Mycophenolic acid	367 (90.9)
Azathioprine	1 (0.2)
CMV prophylaxis in the month prior to QF-CMV determination (yes)	129 (31.9)
Acute graft rejection treated in the previous month (yes)	4 (1.0)
Steroids boluses	2 (0.5)
Plasmapheresis	2 (0.5)
CMV replication in the month prior to QF-CMV determination (yes)	84 (20.8)
Viral load in the month prior to QF-CMV determination, IU/mL, median (IQR)	85.4 (34.5–513.6)
Posttransplant day of QF-CMV determination, median (IQR)	
Kidney transplant	90.0 (60.0–90.0)
Lung transplant	240.0 (150.0–300.0)
QF-CMV value, IU/mL, median (IQR)	
Kidney transplant	4.7 (1.1–18.6)
Lung transplant	10.8 (2.0–42.3)
CMV replication in the month following QF-CMV determination (yes)	90 (22.3)
Viral load in the month following QF-CMV determination, IU/mL, median (IQR)	136.4 (36.0–960.9)
CS-CMV in the month following QF-CMV determination	17 (4.2)
Kidney transplant	12 (3.0)
Lung transplant	5 (1.2)
CMV disease in the month following QF-CMV determination	1 (0.2)
Acute graft rejection treated in the month following QF-CMV determination (yes) ^b^	1 (0.2)

Data are presented as the number of patients (%) unless otherwise indicated. ^a^ All KT recipients received induction therapy with ATG. ^b^ Empirically treated rejection not confirmed by biopsy. ATG, anti-thymocyte globulin; CS-CMV, clinically significant CMV infection; IQR, interquartile range; QF-CMV, QuantiFERON-CMV.

**Table 2:** Characteristics of the study samples (*n* = 404) based on the QF-CMV optimal cutoff value.

	IFN-γ Production in QF-CMV		
Characteristics	<2.2 UI/mL (*n* = 117)	≥2.2 UI/mL (*n* = 287)	*p* Value ^a^
Age, years, median (IQR)	60.0 (55.0–65.0)	61.0 (55.0–65.0)	0.251
Sex			0.306
Male	69 (59.0)	186 (64.8)	
Type of transplant			0.13
Kidney transplant	46 (39.3)	89 (31.0)	
Lung transplant	71 (60.7)	198 (69.0)	
Induction therapy			0.195
ATG	46 (39.3)	89 (31.0)	
Basiliximab	56 (47.9)	146 (50.9)	
Primary immunosuppression regimen			
mTOR inhibitor (Everolimus)	2 (1.7)	12 (4.18)	0.367
Mycophenolic acid	108 (92.3)	259 (90.2)	0.644
Azathioprine	0 (0.0)	1(0.3)	1
CMV prophylaxis in the month prior to QF-CMV determination (yes)	47 (40.2)	82 (28.6)	0.076
Acute graft rejection treated with steroid boluses in the previous month	0 (0.0)	2 (0.7)	1
CMV replication in the month prior to QF-CMV determination (yes)	29 (24.8)	55 (19.2)	0.225
Viral load in the month prior to QF-CMV determination, IU/mL, median (IQR)	145.4 (37.9–788.6)	75.0 (34.5–454.5)	0.163
Posttransplant day of QF-CMV determination, median (IQR)	150.0 (90.0–240.0)	180.0 (90.0–270.0)	0.125
CMV replication in the month following QF-CMV determination (yes)	37 (31.6)	53 (18.5)	0.005
Viral load in the month following QF-CMV determination, IU/mL, median (IQR)	189.1 (35.7–3645.0)	136.36 (36.0–513.6)	0.176
CS-CMV in the month following QF-CMV determination	14 (12.0)	3 (1.0)	<0.001

Data are presented as the number of patients (%) unless otherwise indicated. ^a^ The χ^2^ test or the Fisher exact test was used for categorical variables, and Student’s *t* test or the Mann–Whitney U test was used for continuous variables. ATG, anti-thymocyte globulin; CS-CMV, clinically significant CMV infection; IQR, interquartile range; QF-CMV, QuantiFERON-CMV.

**Table 3:** Diagnostic accuracy of the QF-CMV assay at the different positivity thresholds tested for IFN-γ production to predict protection from CS-CMV out to 30 days post-QF-CMV determination. During this period, patients were without antiviral prophylaxis. (a) KT recipients: CMV seropositive receiving ATG induction; (b) LT recipients: CMV seropositive.

**(a)**					
**Cutoff value (CMV minus nil) IU/mL ^a^**	**Proportion of samples (%)**	**Sensitivity** **(%[95%CI])**	**Specificity** **(%[95%CI])**	**PPV** **(%[95%CI])**	**NPV** **(%[95%CI])**
INF-γ ≥ 0.2 ^b^	91.1	91.0 (86.0–96.0)	8.0 (7.3–23.0)	91.0 (86.0–96.0)	8.0 (0.0–23.3)
INF-γ ≥ 2.2 ^c^	65.2	72.0 (64.2–79.8)	92.0 (84.0–100.0)	99.0 (98.0–100.0)	24.0 (11.8–36.2)
**(b)**					
**Cutoff value (CMV minus nil) IU/mL ^a^**	**Proportion of samples (%)**	**Sensitivity** **(%[95%CI])**	**Specificity** **(%[95%CI])**	**PPV** **(%[95%CI])**	**NPV** **(%[95%CI])**
INF-γ ≥ 0.2 ^b^	94.4	94.0 (91.0–96.8)	0.0	98.0 (96.3–99.7)	0.0
INF-γ ≥ 2.2 ^c^	73.6	75.0 (69.8–80.2)	80.0 (45.0–100.0)	99.5 (98.0–100.0)	5.7 (0.6–10.8)

^a^ Nonreactive and indeterminate results were jointly considered as nonreactive (0.0 IU/mL). ^b^ Cutoff value provided by the manufacturer. ^c^ Optimal cutoff value using Youden’s Index [27]. ATG, anti-thymocyte globulin; CS-CMV, clinically significant CMV infection; KT, kidney transplant; LT, lung transplant; NPV, negative predictive value; PPV, positive predictive value; QF-CMV, QuantiFERON-CMV.

**Table 4:** Quanti-CMV predictive score of the risk of developing clinically significant CMV infection in the month following QF-CMV determination.

Variable	Beta Coefficient	Score
QF-CMV determination <2.2 IU/mL	2.10	3
Induction therapy with ATG	1.40	2
CMV replication the month prior to QF-CMV determination	1.28	2
Total		7

ATG, anti-thymocyte globulin; CMV, cytomegalovirus; QF-CMV, QuantiFERON-CMV.

**Table 5:** Diagnostic accuracy of the Quanti-CMV score to predict the risk of clinically significant CMV infection out to 30 days post-QF-CMV determination in CMV-seropositive KT and LT recipients without antiviral prophylaxis.

Score	Proportion of Samples (%)	Sensitivity (%[95%CI])	Specificity (%[95%CI])	PPV (%[95%CI])	NPV (%[95%CI])	Accuracy (%[95%CI])
Score ≥ 0	100.0	100.0	0.0	4.2 (2.3–6.1)	0.0	4.2 (0.0–13.8)
Score ≥ 2	58.7	88.2 (68.0–100.0)	42.6 (37.2–46.8)	6.3 (3.3–9.0)	98.8 (97.2–100.0)	44.3 (39.5–49.1)
Score ≥ 3	34.9	88.2 (68.0–100.0)	67·4 (62.8–72.0)	10.6 (5.6–15.6)	99.2 (98.1–100.0)	67.8 (63.2–72.3)
Score ≥ 4	20.8	82.4 (64.3–100.0)	81.9 (78.1–85.7)	16.7 (8.7–24.7)	99.1 (98.0–100.0)	81.7 (77.9–85.5)
Score ≥ 5	14.9	76.5 (56.3–96.6)	87.9 (84.8–91.1)	21.7 (11.3–32.1)	98.8 (97.7–99.9)	87.1 (83.8–90.4)
Score = 7	3.7	35.3 (12.6–58.0)	97.7 (96.2–99.1)	40.0 (15.2–64.8)	97.2 (95.6–98.8)	95.0 (92.9–97.1)

KT, kidney transplant; LT, lung transplant; NPV, negative predictive value; PPV, positive predictive value; QF-CMV, QuantiFERON-CMV.

## Data Availability

After de-identification, individual participant data, such as the study protocol, statistical analysis plan, and analytic code, that underlie the results reported in this article can be made available. These data will be available immediately following publication, with no end date, to researchers who provide a methodologically sound proposal to achieve the approved proposal’s aims. Proposals should be directed to elisa.ruiz.arabi.sspa@juntadeandalucia.es; data requestors must sign a data access agreement to gain access.

## Supplementary Materials

The following supporting information can be downloaded at https://www.mdpi.com/article/10.3390/microorganisms13030589/s1, Table S1: univariate logistic regression analysis; Figure S1: cumulative hazard curves of clinically significant CMV infection according to different criteria for QF-CMV assay positivity; Figure S2: posttransplant kinetics of QF-CMV values; Figure S3: Classification and Regression Tree (CART) analysis; Figure S4: cumulative hazard curves of clinically significant CMV infection according to the Quanti-CMV score: <3 and ≥3.

## Abbreviations

The following abbreviations are used in this manuscript:

ATG	antithymocyte globulin
AUROC	area under the receiver operating characteristics curve
CART	Classification and Regression Tree
CI	confidence interval
CMI	cell-mediated immunity
CMV	cytomegalovirus
CMV-CMI	CMV-specific cell-mediated immunity
CS-CMV	clinically significant CMV infection
D	donor
ELISA	enzyme-linked immunosorbent assays
ELISpot	enzyme-linked immunosorbent spot assays
HIV	human immunodeficiency virus
HLA	human leukocyte antigen
ICS	intracellular cytokine staining
IFN-γ	interferon-gamma
IGRA	interferon-γ release assay
IQR	interquartile range
KT	kidney transplant
LT	lung transplant
NPV	negative predictive value
PCR	polymerase chain reaction
PPV	positive predictive value
QNAT	quantitative nucleic acid amplification test
QF-CMV	QuantiFERON-CMV
R	recipient
RCT	randomized controlled trial
RT-PCR	real-time polymerase chain reaction
SE	standard error
SOT	solid organ transplantation
