# Supplementary material for: Quantifying Gamma-Interferon from CMV-Specific CD8+ T Cells Defines Protection Against Clinically Significant CMV Infection in Solid Organ Transplant Recipients: The Quanti-CMV Score
Source: Microorganisms. 2025 Mar 4;13(3):589. doi: 10.3390/microorganisms13030589 (PMC11944424; doi:10.3390/microorganisms13030589)

Supplemental material

Table of contents

1. Tables

TableS1. Univariate logistic regression analysis ………………………………….... page 2

2. Figures

Figure S1. Cumulative hazard curves of clinically significant CMV infection according to different criteria for QF-CMV assay positivity ……………………………..……. page 3-6

Figure S2. Posttransplant kinetics of QF-CMV values ….…………………..……. page 7-8

Figure S3. Classification and Regression Tree (CART) analysis ……………..…….. page 9

Figure S4. Cumulative hazard curves of clinically significant CMV infection according to the Quanti-CMV score: <3 and ≥3 ………………………………………….……... page 10

1. Tables

Table S1. Univariate logistic regression analysis for developing clinically significant CMV infection.

Variable	OR (95%CI)	*P* Value
Age	1.00 (0.95-1.04)	0.97
Male sex	0.83 (0.31-2.22)	0.71
Kidney Transplant[a]	5.15 (1.78-14.90)	0.003
QF-CMV determination ≥ 2.2	0.08 (0.02-0.28)	<0.001
Posttransplant day > 225	0.11 (0.01-0.84)	0.03
Induction therapy with ATG[a]	5.15 (1.78-14.90)	0.003
CMV prophylaxis in the month prior to QF-CMV determination	1.64 (0.55-4.88)	0.37
CMV replication the previous month	4.68 (1.75-12.50)	0.002

[a]All KT recipients received induction therapy with ATG

ATG, anti-thymocyte globulin; CMV, cytomegalovirus; KT, kidney transplant; OR, odds ratio; QF-CMV, QuantiFERON-CMV.

2. Figures

Figure S1. Cumulative hazard curves of clinically significant CMV infection according to different criteria for QF-CMV assay positivity based on IFN-γ levels (CMV minus nil): (a) cutoff value proposed by the manufacturer (≥0.2 IU/mL) in KT recipients (log-rank *P* = 0.83); (b) optimal cutoff value (≥2.2 IU/mL) in KT recipients (log-rank *P* < 0.001); (c) cutoff value proposed by the manufacturer (≥0.2 IU/mL) in LT recipients (log-rank *P* = 0.59); (d) optimal cutoff value (≥2.2 IU/mL) in LT recipients (log-rank *P* = 0.006). QF quantification of CMV < 0.2 IU/mL and <2.2 IU/mL includes nonreactive and indeterminate results.

CMV, cytomegalovirus; IFN-γ: interferon-gamma; KT, kidney transplant; LT, lung transplant; QF-CMV, QuantiFERON-CMV.

a

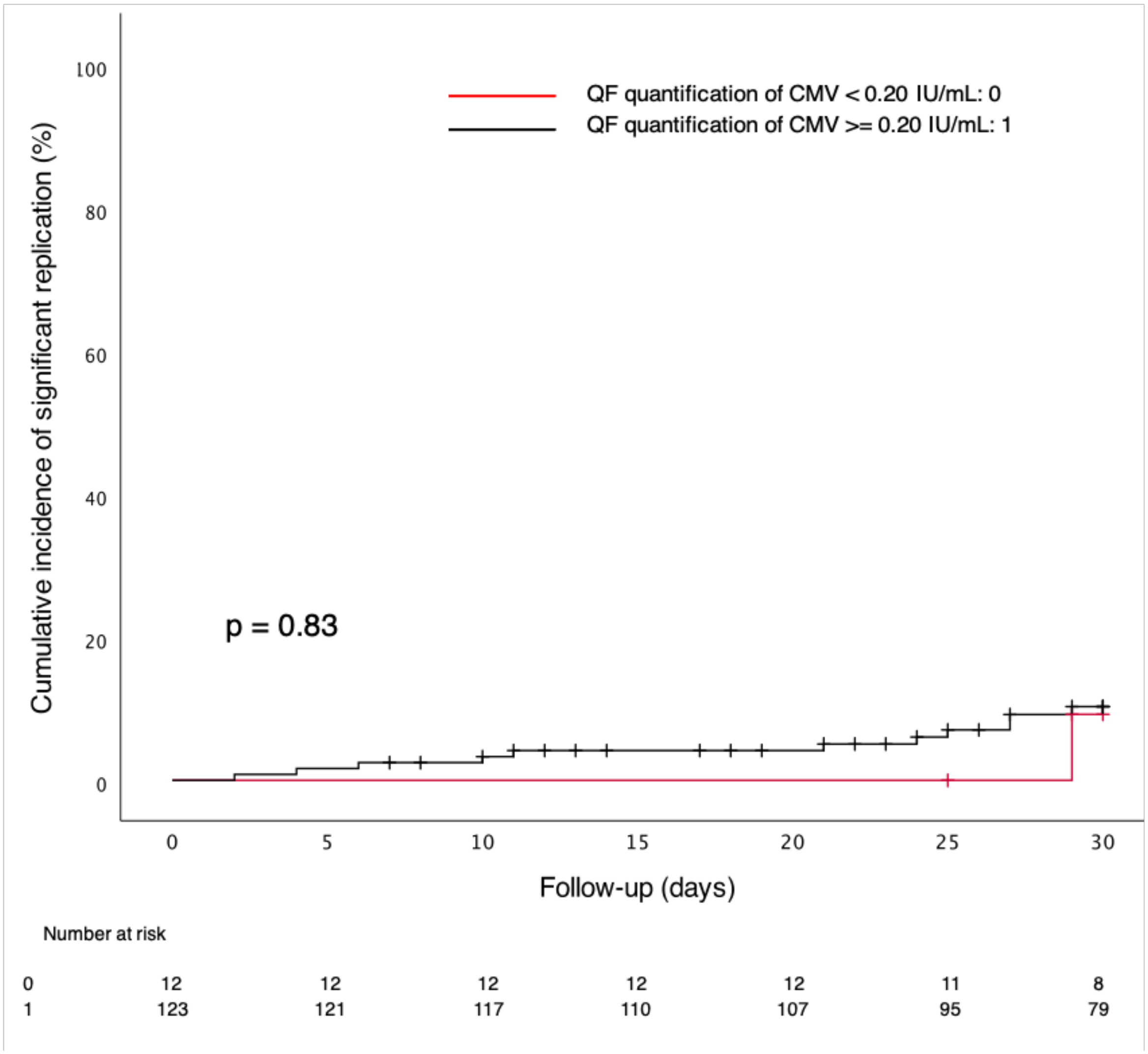

b

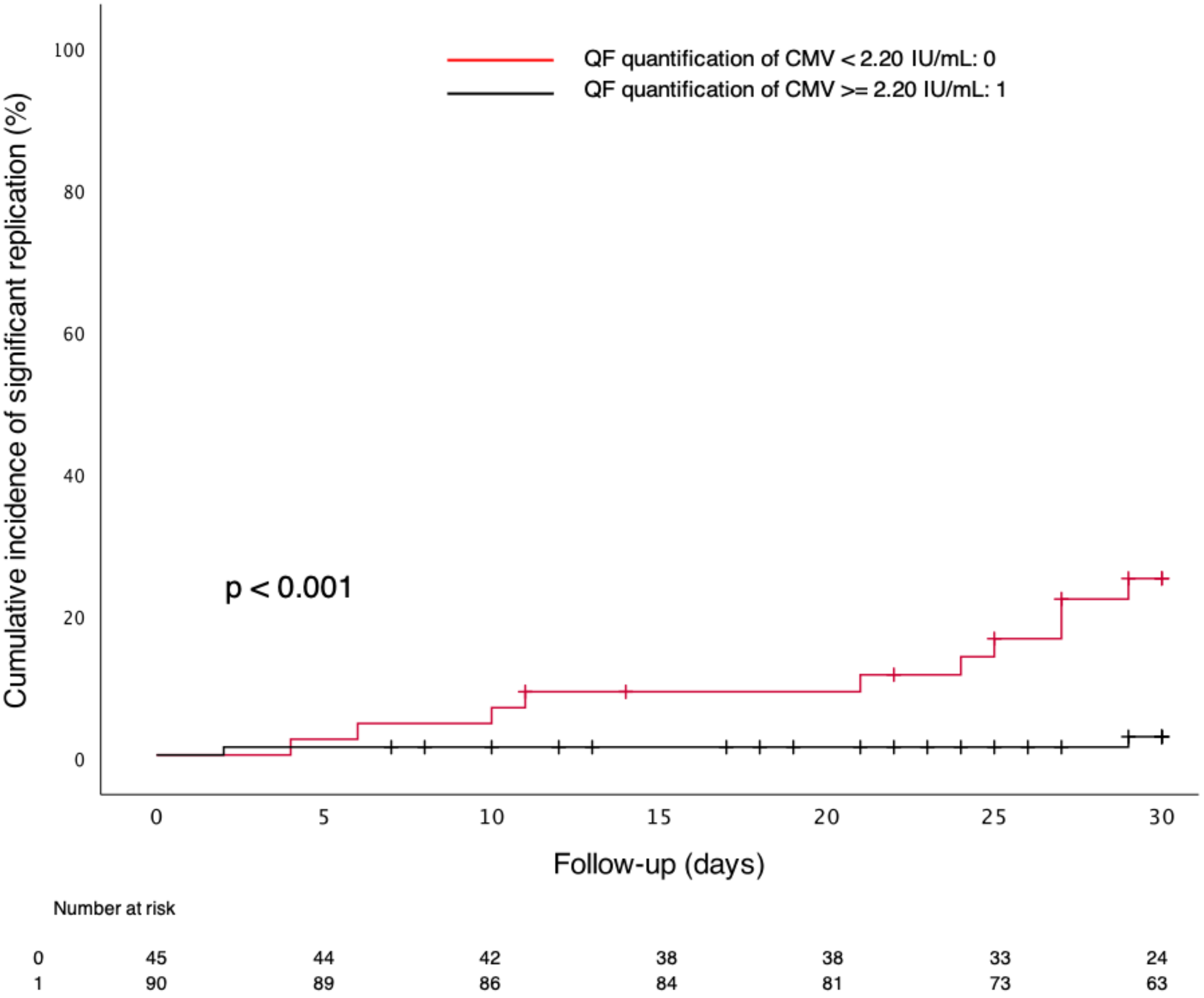

QF quantification of CMV < 2.20 IU/mL: 0
QF quantification of CMV >= 2.20 IU/mL: 1
Cumulative incidence of significant replication (%)
100
80
60
40
20
0
p < 0.001
0
5
10
15
20
25
30
Follow-up (days)
Number at risk
0 45 44 42 38 38 33 24
1 90 89 86 84 81 73 63

c

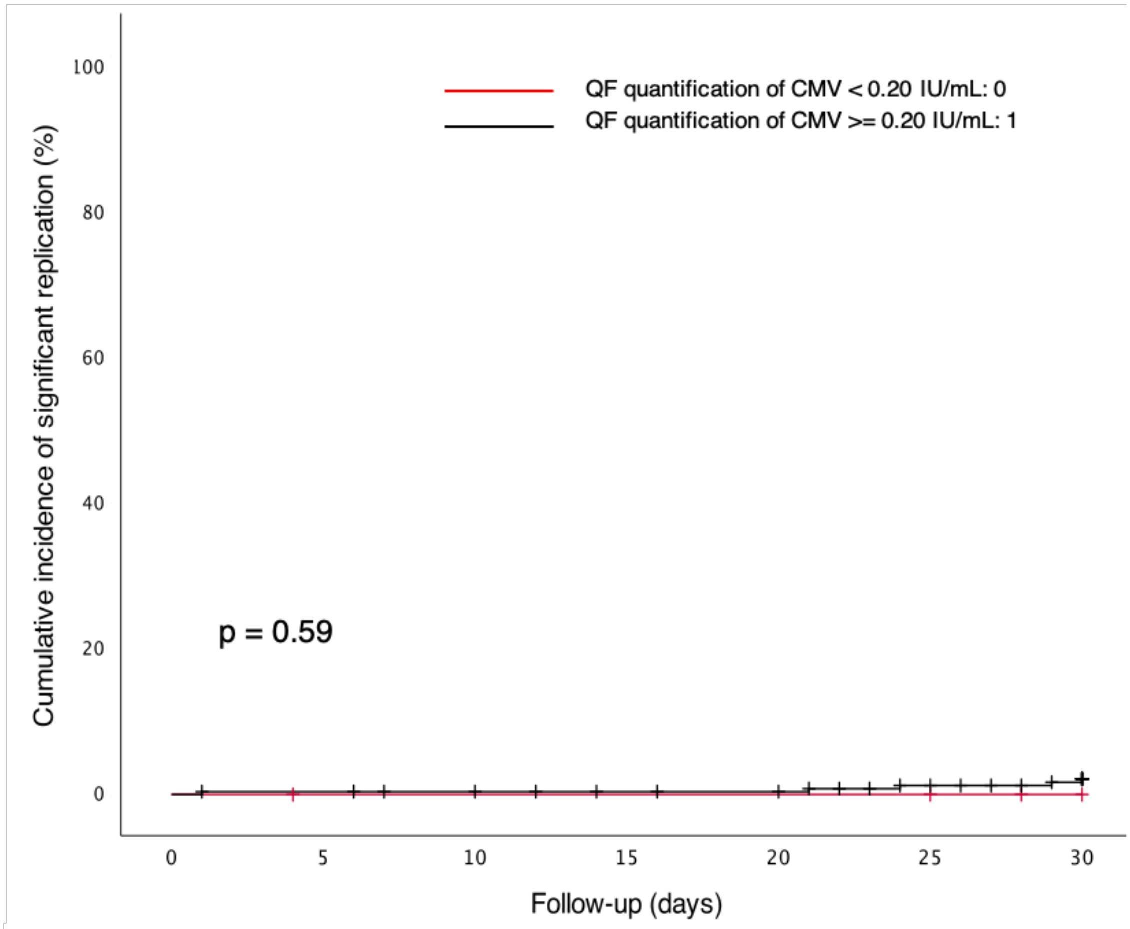

Number at risk

0	15	14	14	14	14	13	11
1	254	252	249	247	245	231	220

d

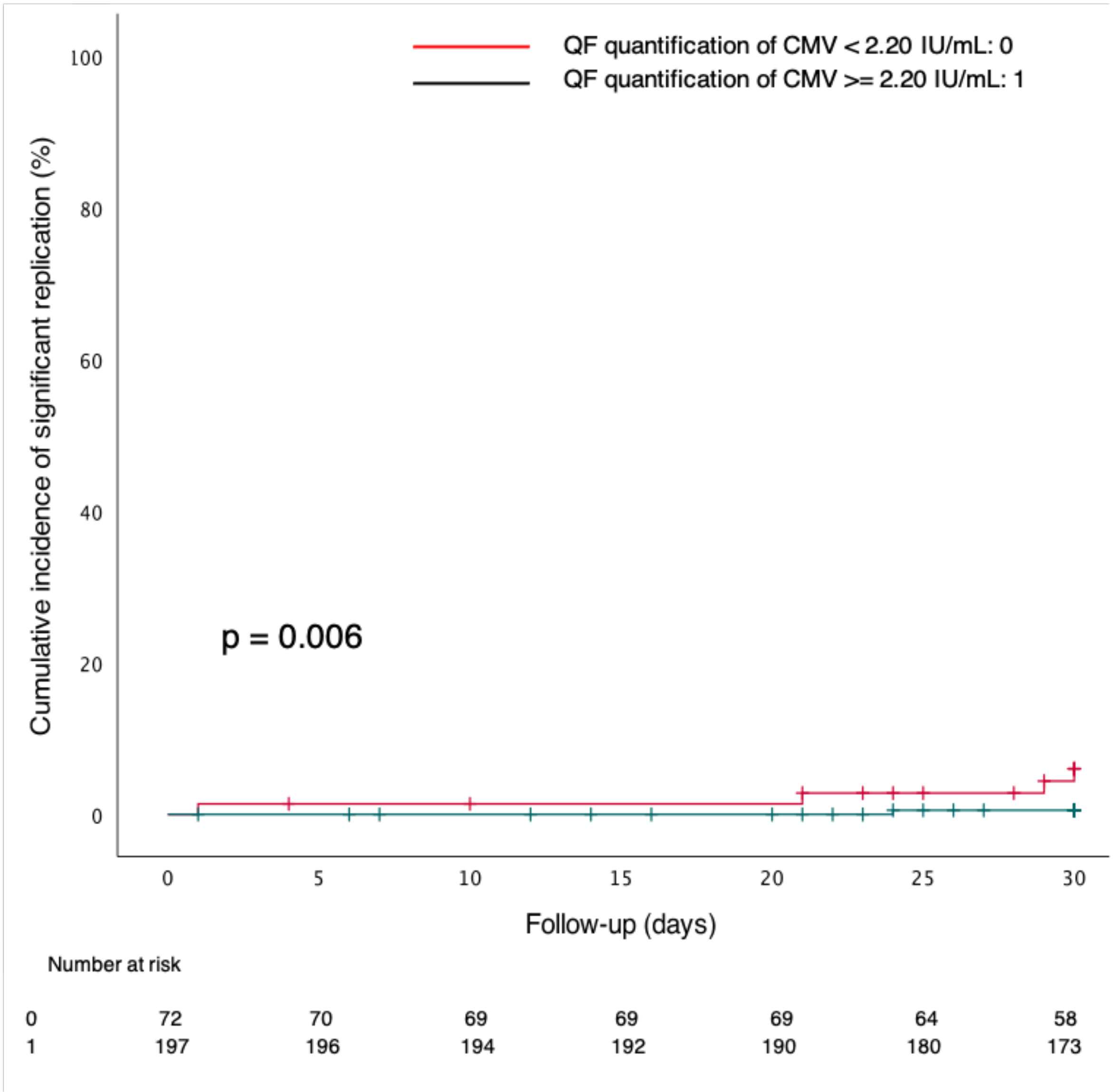
QF quantification of CMV < 2.20 IU/mL: 0
QF quantification of CMV >= 2.20 IU/mL: 1
Cumulative incidence of significant replication (%)
100
80
60
40
20
0
p = 0.006
0
5
10
15
20
25
30
Follow-up (days)
Number at risk
0 72 70 69 69 69 64 58
1 197 196 194 192 190 180 173

Figure S2. Posttransplant kinetics of QF-CMV values expressed as continuous variables. (a) LT recipients; (b) KT recipients. Values are shown as individual diamonds; horizontal lines represent median values, limits of the boxes represent interquartile ranges, and whiskers represent maximum and minimum values (extreme values). Beyond the whiskers are the outliers.

KT: kidney transplant; LT: lung transplant; QF-CMV: QuantiFERON-CMV

a

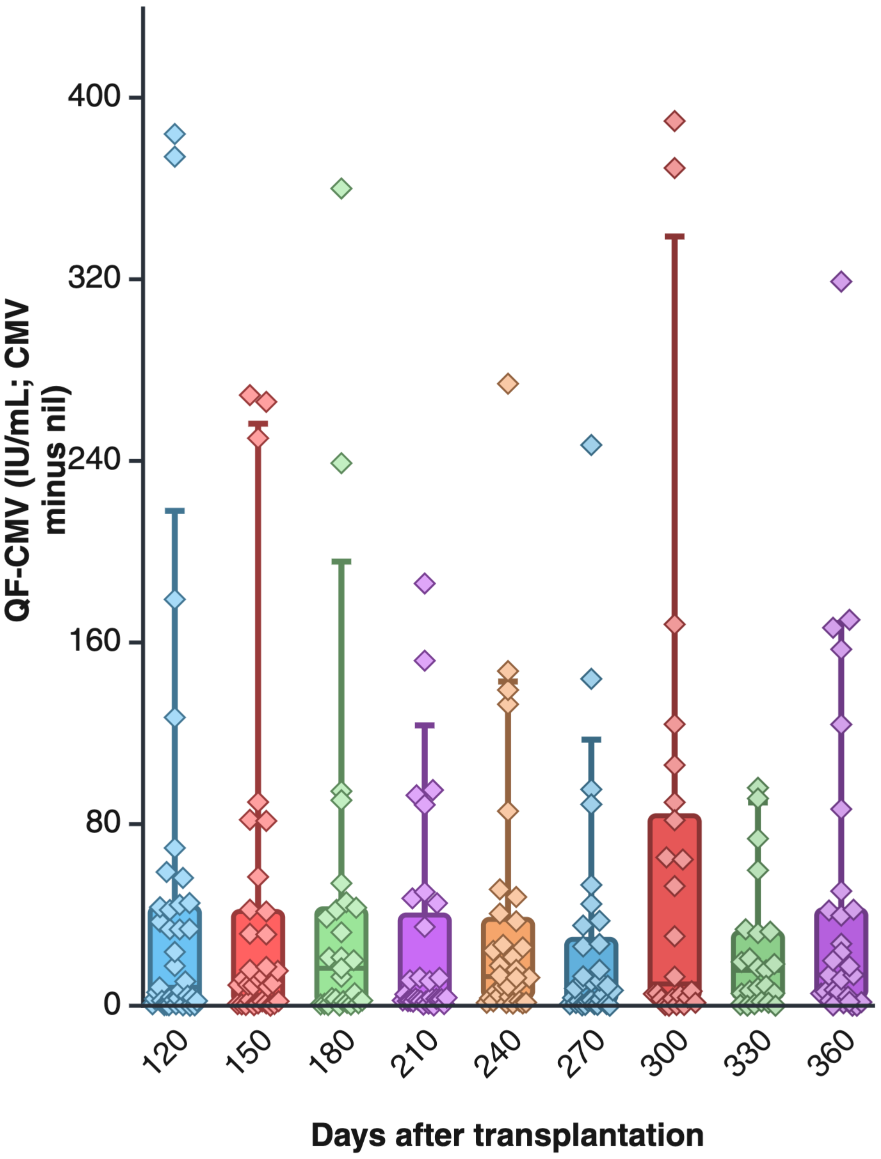

b

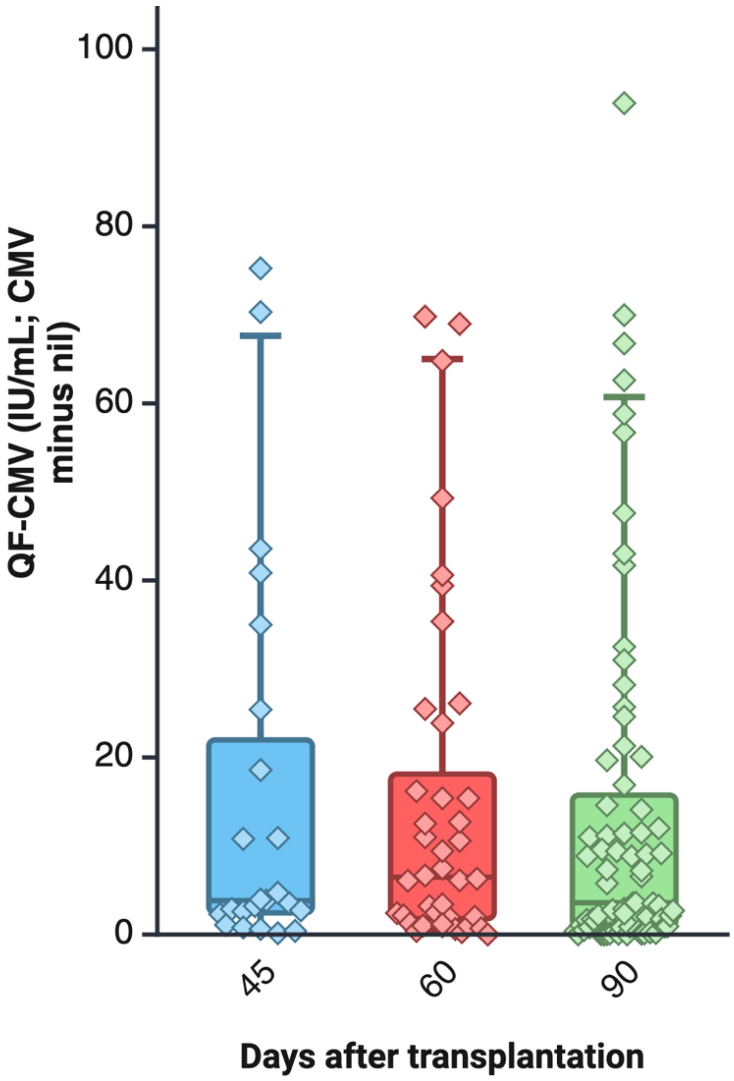

*Created with BioRender.com

Figure S3. Classification and Regression Tree (CART) analysis identifying predictive variables for clinically significant CMV infection out to 30 days post-QF-CMV determination. Predictive variables are QF-CMV ≥ 2.2 IU/mL (yes/no), induction therapy with ATG (yes/no), and CMV replication in the month before QF-CMV determination (yes/no).

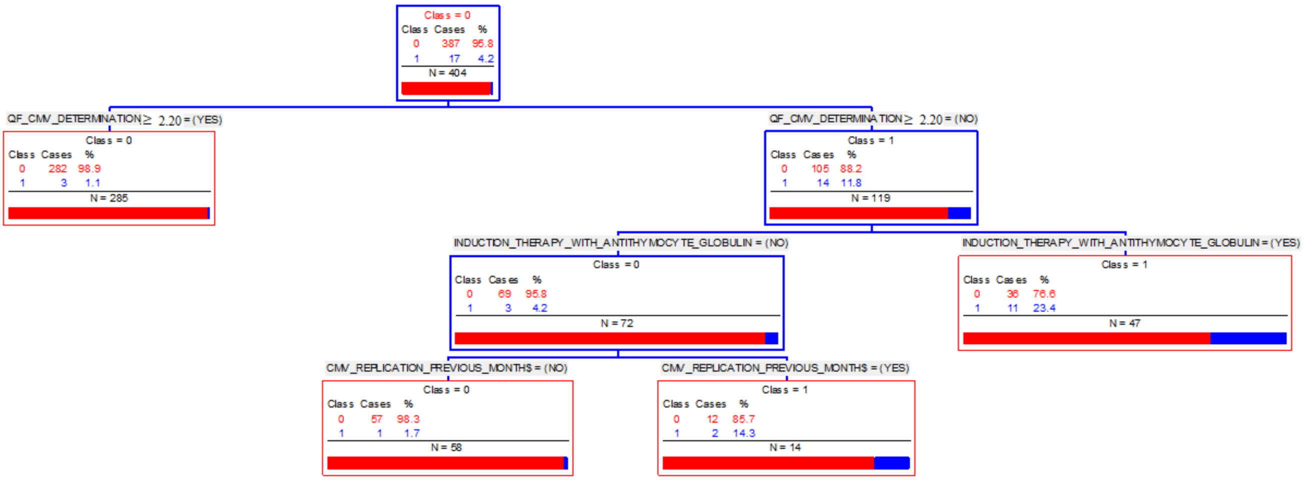

ATG, anti-thymocyte globulin; CMV, cytomegalovirus; QF-CMV, QuantiFERON-CMV

Note: 1: clinically significant CMV infection.
0: no clinically significant CMV infection.

Figure S4. Cumulative hazard curves of clinically significant CMV infection according to the Quanti-CMV score: <3 and ≥3 (log-rank *P* < 0.0001)

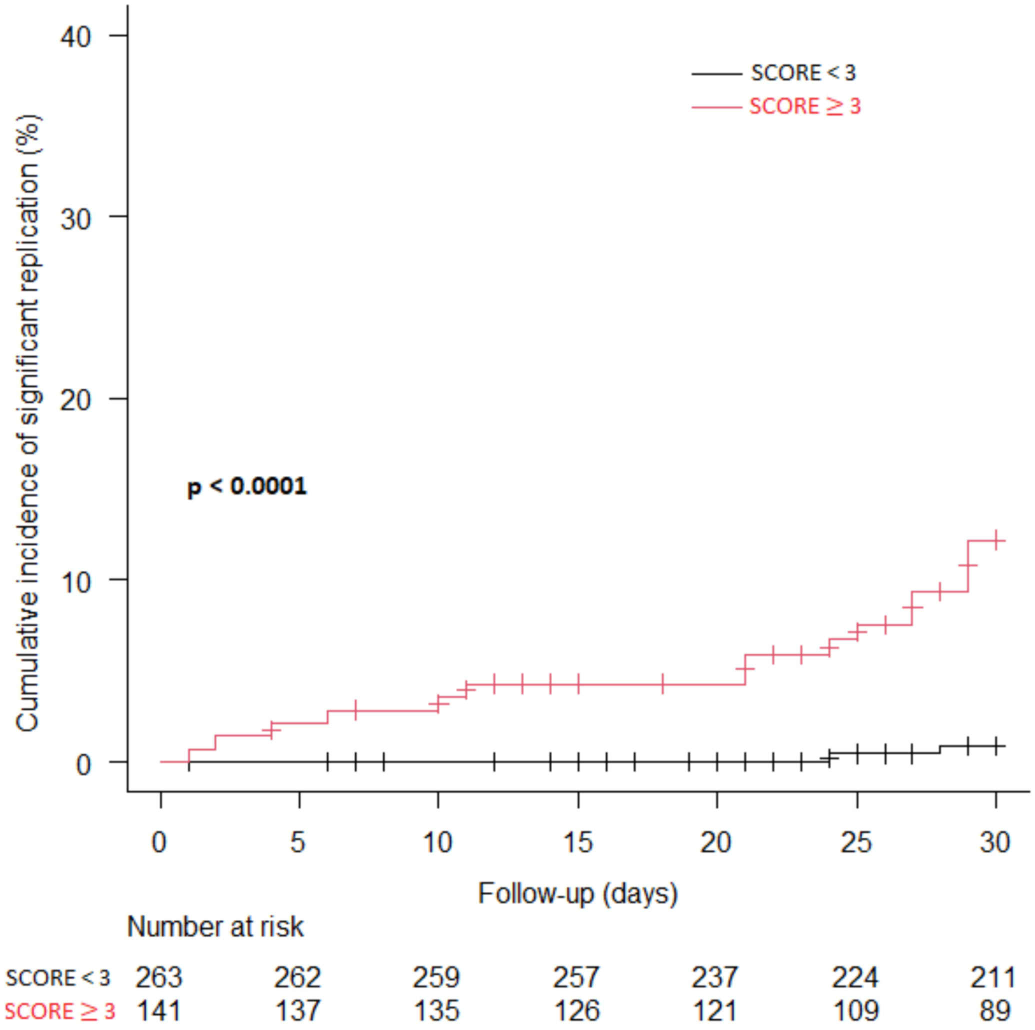

Supplement: Supplementary file 1 [file microorganisms-13-00589-s001.zip › microorganisms-3507602-supplementary.pdf]
